# Optogenetic evocation of field inhibitory postsynaptic potentials in hippocampal slices: a simple and reliable approach for studying pharmacological effects on GABA_A_ and GABA_B_ receptor-mediated neurotransmission

**DOI:** 10.3389/fncel.2014.00002

**Published:** 2014-01-22

**Authors:** Julien Dine, Claudia Kühne, Jan M. Deussing, Matthias Eder

**Affiliations:** ^1^Research Group Neuronal Network Dynamics, Max Planck Institute of PsychiatryMunich, Germany; ^2^Research Group Molecular Neurogenetics, Max Planck Institute of PsychiatryMunich, Germany

**Keywords:** optogenetics, hippocampus, field inhibitory postsynaptic potential, GABA receptor, channelrhodopsin, CA1, neuroactive steroid, extracellular recording

## Abstract

The GABAergic system is the main source of inhibition in the mammalian brain. Consequently, much effort is still made to develop new modulators of GABAergic synaptic transmission. In contrast to glutamatergic postsynaptic potentials (PSPs), accurate monitoring of GABA receptor-mediated PSPs (GABAR-PSPs) and their pharmacological modulation in brain tissue invariably requires the use of intracellular recording techniques. However, these techniques are expensive, time- and labor-consuming, and, in case of the frequently employed whole-cell patch-clamp configuration, impact on intracellular ion concentrations, signaling cascades, and pH buffering systems. Here, we describe a novel approach to circumvent these drawbacks. In particular, we demonstrate in mouse hippocampal slices that selective optogenetic activation of interneurons leads to prominent field inhibitory GABA_A_R- and GABA_B_R-PSPs in area CA1 which are easily and reliably detectable by a single extracellular recording electrode. The field PSPs exhibit typical temporal and pharmacological characteristics, display pronounced paired-pulse depression, and remain stable over many consecutive evocations. Additionally validating the methodological value of this approach, we further show that the neuroactive steroid 5α-THDOC (5 μM) shifts the inhibitory GABA_A_R-PSPs towards excitatory ones.

## Introduction

In the mammalian brain, the GABAergic system is a key regulator of neuronal activity. Mediated by ionotropic GABA_A_ and metabotropic GABA_B_ receptors (GABA_A_Rs and GABA_B_Rs), inhibitory postsynaptic potentials (PSPs) hyperpolarize neurons, thereby attenuating action potential (AP) firing. An imbalance between neuronal excitation and inhibition often results in severe neurological and psychiatric conditions (e.g., epilepsy and psychosis). This fact still prompts researchers to develop novel modulators of GABAergic neurotransmission (Enna and Möhler, 2007).

A state-of-the-art approach to probe a particular substance for modulatory actions on the GABAergic system is to investigate its impact on GABA_A_R- and/or GABA_B_R-mediated PSPs or currents in acute brain slice preparations (Whittington et al., 1995; Nugent et al., 2007). However, the mostly diffuse distribution of GABAergic cells in neuronal networks and their predominantly local axonal arborizations (DeFelipe et al., 2013) makes it nearly impossible to reliably and non-laboriously detect prominent (electrically evoked or spontaneous) GABA receptor-mediated PSPs (GABAR-PSPs) by means of a single extracellular recording electrode (Bazelot et al., 2010). In contrast, this is for instance easily achievable for glutamatergic PSPs in hippocampal slices (Bliss and Collingridge, 1993; Stepan et al., 2012). Therefore, intracellular recording techniques still represent the gold standard to uncover pharmacological effects on GABAR-PSPs. Yet, these methods have some drawbacks. First, they are quite expensive and time- and labor-consuming. Second, stable long-term recordings cannot reliably be achieved. And third, the widely used whole-cell patch-clamp technique affects intracellular ion concentrations, signaling cascades, and pH buffering systems (Burg et al., 1998; Eder et al., 2002; Nugent et al., 2007). This is for instance critical for GABA_A_R-PSPs since the reversal potential for chloride ions lies close to the resting membrane potential (RMP) of neurons (Bormann, 1988; Eder et al., 2001). Furthermore, it has been shown that natural pH buffering systems can be crucial for the impact of a particular neuroactive factor on GABAergic neurotransmission (Burg et al., 1998).

Developed in the recent past, optogenetic tools allow one to endow neurons with the light-sensitive Channelrhodopsin-2 (ChR2). By activating this excitatory opsin, it is possible to elicit APs in the expressing cells with millisecond precision (Boyden et al., 2005; Madisen et al., 2012). Hippocampal CA1 pyramidal cells are strongly innervated in the somatic and perisomatic region by nearby interneurons (especially basket cells; Andersen et al., 2007). Therefore, we examined whether blue light pulses delivered to area CA1 in brain slices from mice selectively expressing Channelrhodopsin-2 (ChR2) in GABAergic neurons (“GABA-ChR2 mice”) can evoke pronounced field GABAR-PSPs which are reliably detectable by an extracellular recording pipette (positioned close to the CA1 cell body layer). Since this was the case, we further explored, among other things, temporal and pharmacological characteristics of the field GABAR-PSPs, their stability over time, and the impact of the neuroactive steroid 5α-THDOC (Burg et al., 1998) on GABA_A_R-PSPs. The results of these investigations validate the optogenetic approach employed here as a potentially useful tool for future studies dealing with pharmacological manipulations of the GABAergic system.

## Materials and methods

### Animals

For all experiments, 4-month-old male mice were used. Experiments were approved by the Committee on Animal Health and Care of the local governmental body and performed in compliance with the guidelines for the care and use of laboratory animals set by the European Community. Mice selectively expressing ChR2(H134R)-EYFP in forebrain GABAergic neurons (“GABA-ChR2 mice”) were generated by breeding hemizygous *Dlx5/6-Cre* mice (Refojo et al., 2011) to homozygous Ai32 mice (purchased from the Jackson Laboratory; Madisen et al., 2012). Generation of *Dlx5/6-Cre* mice and their spatial pattern of Cre activity have been described in detail (Monory et al., 2006). Briefly, the vector for transgenic expression of Cre recombinase was constructed as follows: a 1.4 kb EcoRI-XhoI fragment of the zebrafish *dlx5a/dlx6a* locus, which recapitulates with a high degree of precision the endogenous *Dlx5* expression patterns in transgenic mice, was placed into a plasmid containing the Cre coding sequence downstream of a 3.5 kb fragment from the immediate 5′-flanking region of zebrafish *dlx6a*, including part of the 5′UTR. The *dlx6a* upstream fragment does not, by itself, produce any tissue-specific expression in transgenic animals, but was shown to increase the activity of the *Dlx5/Dlx6* intergenic enhancers. Genotyping was performed using the following primers specific for *Dlx5/6-Cre*: Dlx-fwd 5′-CAC-GTT-GTC-ATT-GGT-GTT-AG-3′, Dlx-rev 5′-CCG-GTC-ATG-ATG-TTT-TAT-CT-3′, Thy1-F1 5′-TCT-GAG-TGG-CAA-AGG-ACC-TTA-GG 3′, and Thy1-R1 5′-CCA-CTG-GTG-AGG-TTG-AGG-3′. Standard PCR conditions resulted in a Cre-specific PCR product of 313-bp and a control PCR product of 372-bp. Genotyping of Ai9 and Ai32 mice was conducted according to the genotyping protocols provided by the Jackson Laboratory. C57BL/6N mice were obtained from the Max Planck Institute’s breeding colony. The animals were housed under standard laboratory conditions with food and water ad libitum.

### Double *in situ* hybridization

Double *in situ* hybridization of brain sections of *Dlx5/6-Cre; Ai9* mice was performed as previously described using specific probes for *tomato*, murine *Gad65*, and murine *Gad67* (Refojo et al., 2011). Details on riboprobes are available upon request.

### Preparation of brain slices

Mice were anesthetized with isoflurane and decapitated. All following steps were done in ice-cold cutting saline saturated with carbogen gas (95% O_2_/5% CO_2_). This saline (pH 7.4) consisted of (in mM): 125 NaCl, 2.5 KCl, 25 NaHCO_3_, 1.25 NaH_2_PO_4_, 0.5 CaCl_2_, 6 MgCl_2_, and 25 glucose. After decapitation, the brain was rapidly removed from the cranial cavity and 350-μm-thick coronal slices containing the hippocampus were cut using a vibratome (HM650V; Thermo Scientific). Afterwards, slices were incubated for 30 min in carbogenated physiological saline at 34°C. This saline (pH 7.4) consisted of (in mM): 125 NaCl, 2.5 KCl, 25 NaHCO_3_, 1.25 NaH_2_PO_4_, 2 CaCl_2_, 1 MgCl_2_, and 25 glucose. Subsequently, slices were stored at room temperature (23–25°C) for at least 30 or 90 min in carbogenated physiological saline before patch-clamp or field potential recordings, respectively.

### Electrophysiology

All experiments were carried out at room temperature. In the recording chamber, slices were continuously superfused with carbogenated physiological saline (4–5 ml/min flow rate). Field potentials in the transition zone between CA1 stratum pyramidale and CA1 stratum radiatum were recorded using glass microelectrodes (~1 MΩ open-tip resistance, filled with physiological saline) that were connected to an extracellular amplifier (EXT-01, npi electronic). Recording data were low-pass filtered at 500 Hz and digitized at 2.5 kHz. For patch-clamp recordings, individual neurons in area CA1 were visualized by infrared videomicroscopy (Ranft et al., 2007). Somatic whole-cell patch-clamp recordings from CA1 interneurons and pyramidal cells (>1 GΩ seal resistance) were performed in bridge or voltage-clamp mode using a SEC-10L amplifier (npi electronic). The potential/current was low-pass filtered at 1.3 kHz and digitized at 6.5 kHz. The patch-clamp electrodes (5–7 MΩ open-tip resistance) were pulled from borosilicate glass capillaries and filled with a solution consisting of (in mM): 130 K-gluconate, 5 NaCl, 2 MgCl_2_, 2 Mg-ATP, 20 phosphocreatine, 0.3 GTP, 10 HEPES, 0.5 EGTA, 5 glucose (pH 7.2, adjusted with KOH). The access resistance (*R*_a_) was continuously monitored. Recordings were terminated if *R*_a_ changed >10%. All potentials were corrected for a liquid junction potential of 12 mV. For extracellular electrical stimulation, square pulse stimuli (200 μs pulse width) were delivered via a custom-made bipolar tungsten electrode (50 μm pole diameter, ~0.5 MΩ nominal impedance) to the neuronal tissue.

### Light application

The light beam of a Sapphire 488 nm (75 mW max. output power) or Sapphire 561 nm (75 mW max. output power) laser (Coherent) was collimated into an optical fiber (BFL37-200, Thorlabs), which was coupled into the epifluorescence port of a Axioskop 2 FS microscope (Zeiss) (Eder et al., 2003). During the field potential recordings, the light pulses were delivered via a 2.5X objective and during the patch-clamp recordings via a 40X water immersion objective to the neuronal tissue. The resultant light spots in the focus plane had diameters of approximately 1 mm (2.5X objective) and 400 μm (40X objective). The duration of the light pulses (2 ms) was regulated by means of a LS3ZM2 shutter and VCM-D1 shutter driver (Vincent Associates). The light intensity in the focus plane was measured using the PM100 system (Thorlabs).

### Chemicals

AP5, Bicuculline methiodide (BIM), CGP 55845, picrotoxin, NBQX, and TTX citrate were purchased from Abcam, isoflurane from Abbott, and 5α-THDOC and all other substances/salts from Sigma-Aldrich. Drugs were applied by bath application.

### Statistics

Statistical analysis was run in SigmaStat (Systat Software), with significance declared at *p* < 0.05. Data are given as mean ± SEM.

## Results

### Patch-clamp recordings from CA1 interneurons and pyramidal cells in brain slices from GABA-ChR2 mice

**Figure 1:**
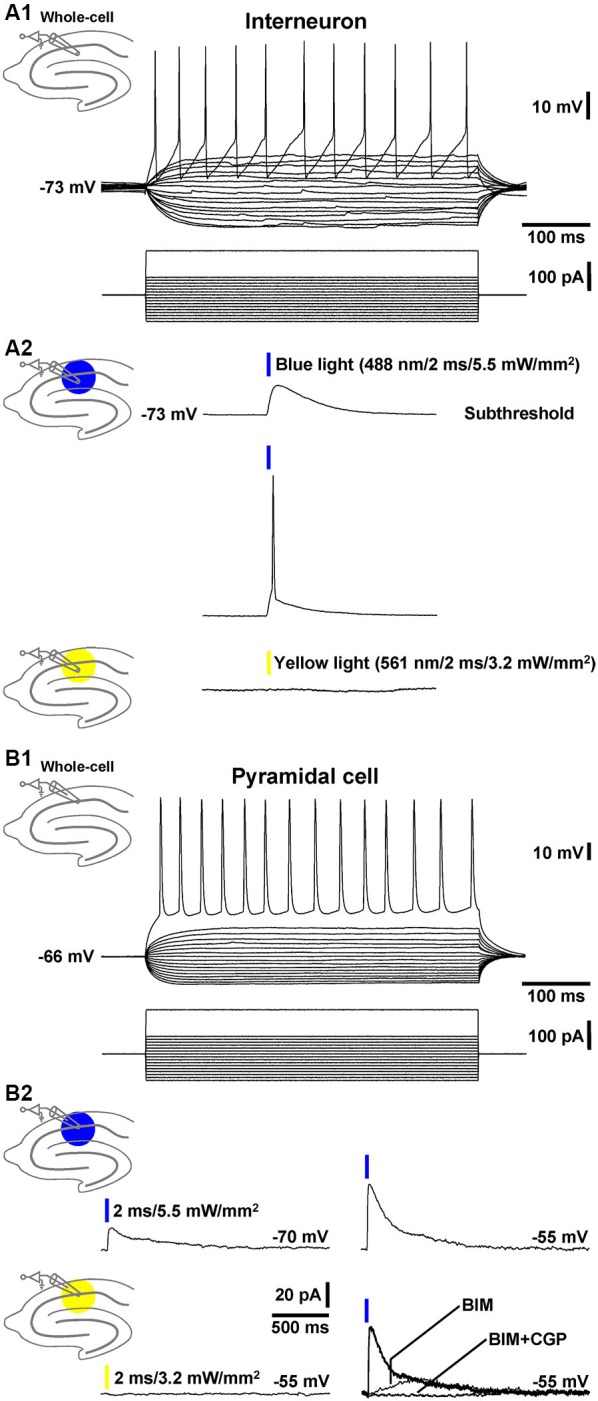
**Whole-cell patch-clamp recordings from a CA1 stratum radiatum cell and pyramidal neuron. (A1)** Current injections into a CA1 stratum radiatum interneuron triggered fast APs followed by prominent afterhyperpolarizations. **(A2)** Blue light pulses delivered to area CA1 evoked subthreshold depolarizations (upper recording trace) and APs (middle recording trace) in the same neuron. Yellow light application failed to induce deflections of the RMP (lower recording trace). **(B1)** APs in a CA1 pyramidal cell triggered by current injections. **(B2)** Blue light pulses delivered to area CA1 elicited inhibitory GABA_A_R- and GABA_B_R-mediated currents (but no excitatory ones) in the same pyramidal neuron. According to the reversal potential for chloride ions, the GABA_A_R-mediated currents showed bigger amplitudes if the membrane potential was clamped from −70 to −55 mV (upper recording traces and right lower panel). Yellow light application failed to induce comparable currents (left lower recording trace). The current traces in the presence of BIM or BIM + CGP 55845 (CGP) were recorded 10 min after the onset of BIM or CGP bath application.

To test whether the transgenic mice generated (GABA-ChR2 mice) express functional ChR2 in GABAergic neurons, we performed whole-cell patch-clamp recordings from stratum radiatum cells that exhibited morphologies and firing characteristics (Figure 1A1) typical for interneurons (Andersen et al., 2007). In all cells recorded (*n* = 4), blue light pulses (488 nm, 2 ms) at the highest attainable intensity (5.5 mW/mm^2^) evoked prominent depolarizations of the RMP, which in most cases (~90%) triggered an AP (Figure 1A2, middle recording trace). As could be expected from the activation spectrum of ChR2 (Zhang et al., 2007), yellow light pulses (561 nm, 2 ms, 3.2 mW/mm^2^) never elicited deflections of the RMP in the same cells (Figure 1A2, lower recording trace).

Next, we recorded from CA1 pyramidal neurons (Figure 1B1) and examined whether application of blue light to area CA1 leads to GABAR-mediated responses in these cells. Indeed, in 3 out of 4 pyramidal neurons under investigation, the light pulses evoked clearly discernable inhibitory currents (Figure 1B2, left upper panel), which increased if the membrane potential was clamped from −70 to −55 mV (Figure 1B2, right upper panel). Yellow light failed to induce such currents (Figure 1B2, left lower panel). The GABA_A_R antagonist BIM (20 µM) blocked the prominent fast component of the inhibitory currents. The remaining slow component was fully abolished by the GABA_B_R antagonist CGP 55845 (5 µM) (Figure 1B2, right lower panel). Altogether, these findings indicate that, in GABA-ChR2 mice, CA1 interneurons which form synapses with CA1 pyramidal cells express functional ChR2 and, upon blue light activation, generate GABA_A_R- and GABA_B_R-PSPs in the pyramidal neurons.

**Figure 2:**
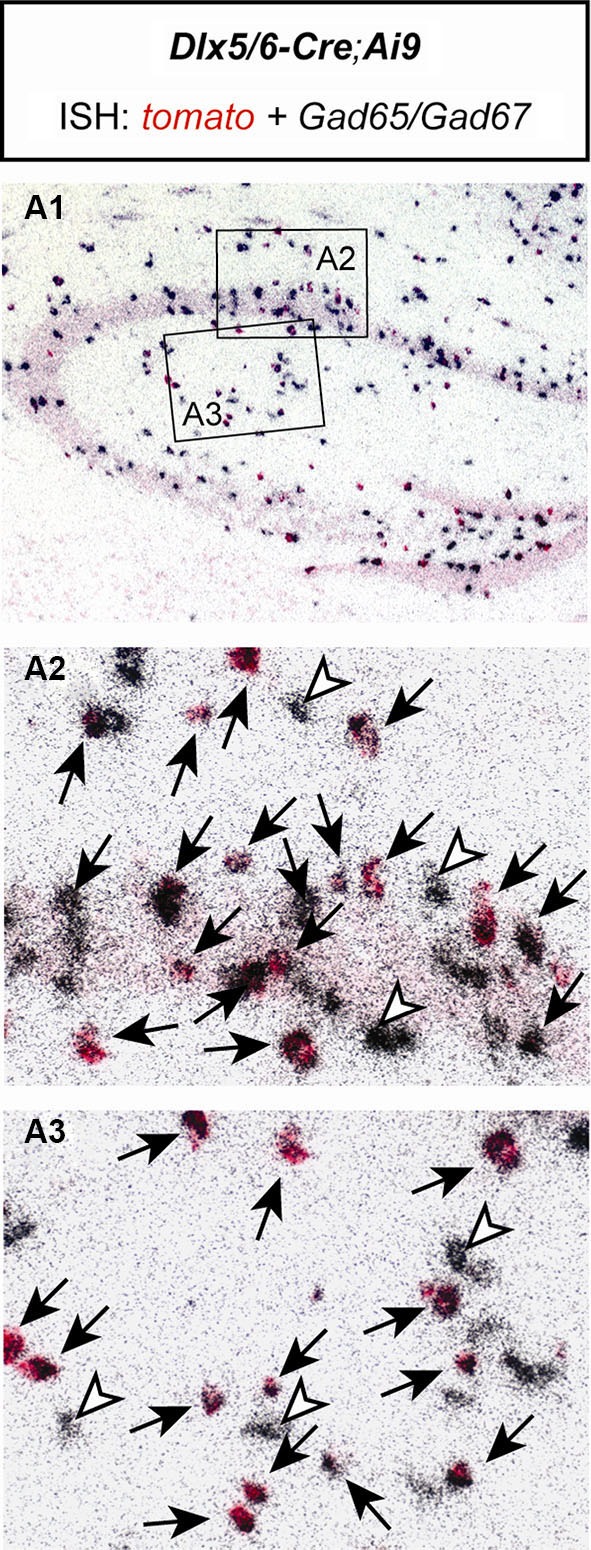
**The Cre recombinase in Dlx5/6-Cre;Ai9** mice activates reporter gene expression exclusively in GABAergic neurons of the hippocampus. Double *in situ* hybridization (ISH) of a brain section of a *Dlx5/6-Cre;Ai9* mouse using specific riboprobes for *tomato* (red) and *Gad65/Gad67* (silver grains, black). **(A1)** Overview of the hippocampus. Respective magnifications of insets are depicted in **(A2)** and **(A3)**. Black arrows indicate cells coexpressing *tomato* and *Gad65/Gad67*. White arrowheads indicate cells expressing only *Gad65/Gad67*.

As an additional test for the specificity of the utilized Cre mouse line, we bred *Dlx5/6-Cre* mice to *Ai9* reporter mice (Madisen et al., 2010). Double *in situ* hybridization of brain sections of *Dlx5/6-Cre;Ai9* mice revealed that all *tomato* expressing cells coexpressed *Gad65/Gad67*. No *Gad65/Gad67* negative cells were detected to express *tomato*. Only very few *Gad65/Gad67* positive cells were detected which did not express *tomato* (Figure 2). These results confirm the previously described exclusive GABAergic identity of Cre-expressing neurons in *Dlx5/6-Cre* mice (Monory et al., 2006; Refojo et al., 2011).

### Field potential recordings in the transition zone between CA1 stratum pyramidale and CA1 stratum radiatum

**Figure 3:**
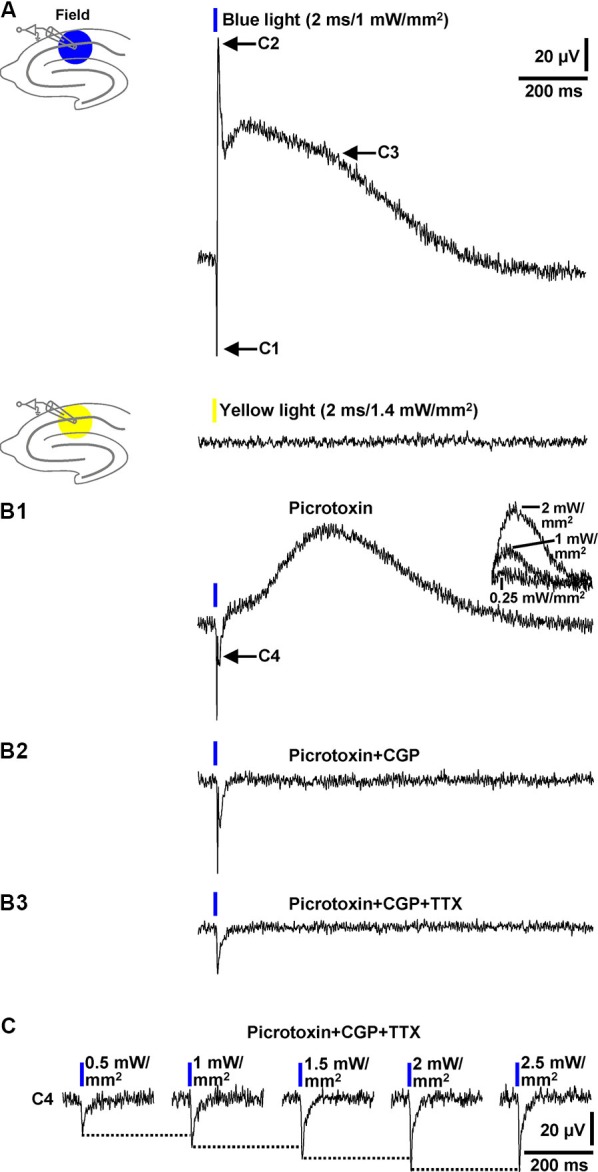
**Field potential recordings in the transition zone between CA1 stratum pyramidale and CA1 stratum radiatum. (A)** Blue (but not yellow) light pulses (≥1 mW/mm^2^) delivered to area CA1 reliably evoked field responses that consisted of three components (C1–C3). Picrotoxin blocked C2 and the initial part of C3 (**B1**), CGP the remaining fraction of C3 (**B2**), and TTX C1 (**B3**). Picrotoxin unmasked a further downwards voltage deflection (C4) (**B1**). The picrotoxin-resistant fraction of C3 could be increased/decreased by use of higher/lower light intensities (**B1**, inset). The voltage traces in the presence of picrotoxin, picrotoxin + CGP, and picrotoxin + CGP + TTX were acquired 20, 5, and 7 min after the onset of picrotoxin, CGP, and TTX administration, respectively. **(C)** C4 increased with increasing intensities (0.5–2.5 mW/mm^2^) of the blue light pulses.

In a next series of experiments, we investigated whether blue light application to area CA1 elicits field inhibitory PSPs which can be detected by an extracellular recording pipette positioned into the transition zone between CA1 stratum pyramidale and CA1 stratum radiatum (without testing different recording sites). In all slices probed (*n* = 13/5 animals), blue light pulses (≥1 mW/mm^2^) triggered field responses that comprised three components. As shown in Figure 3A (upper panel), the first component (C1) represents a fast downwards voltage deflection, which is followed by a fast upwards one (C2). C2 in turn is followed by a slow upwards voltage deflection (C3). Yellow light pulses failed to evoke such field responses (Figure 3A, lower panel). C2 and the initial part of C3 (see Section Discussion) were abolished by picrotoxin (100 µM) [or BIM (20 µM), data not shown] (Figure 3B1), indicating that they are mediated by GABA_A_Rs. This pharmacological treatment unmasked a further downwards voltage deflection (C4). The picrotoxin-resistant fraction of C3, which could be increased/decreased by use of higher/lower light intensities (Figure 3B1, inset), was completely blocked by CGP 55845 (5 µM) (Figure 3B2). Thus, it resulted from an activation of GABA_B_Rs. C1 disappeared in the presence of TTX (1 µM) (Figure 3B3), corroborating our assumption that it reflects APs in interneurons. With regard to the origin of C4, the only plausible explanation is that it arises from the flow of sodium ions through ChR2 (Zhang et al., 2007) into the expressing cells. Consistently, C4 increased with increasing intensities of the light pulses (Figure 3C) and displayed nearly identical kinetics as the subthreshold depolarizations in the interneurons recorded (Figure 1A2, upper recording trace).

Collectively, the data presented in this paragraph show that blue light pulses delivered to area CA1 in brain slices from GABA-ChR2 mice can elicit prominent field inhibitory GABA_A_R- and GABA_B_R-PSPs which are reliably detectable by an extracellular recording electrode placed close to the CA1 cell body layer.

### Quantification, stability, and short-term plasticity of field GABAR-PSPs

**Figure 4:**
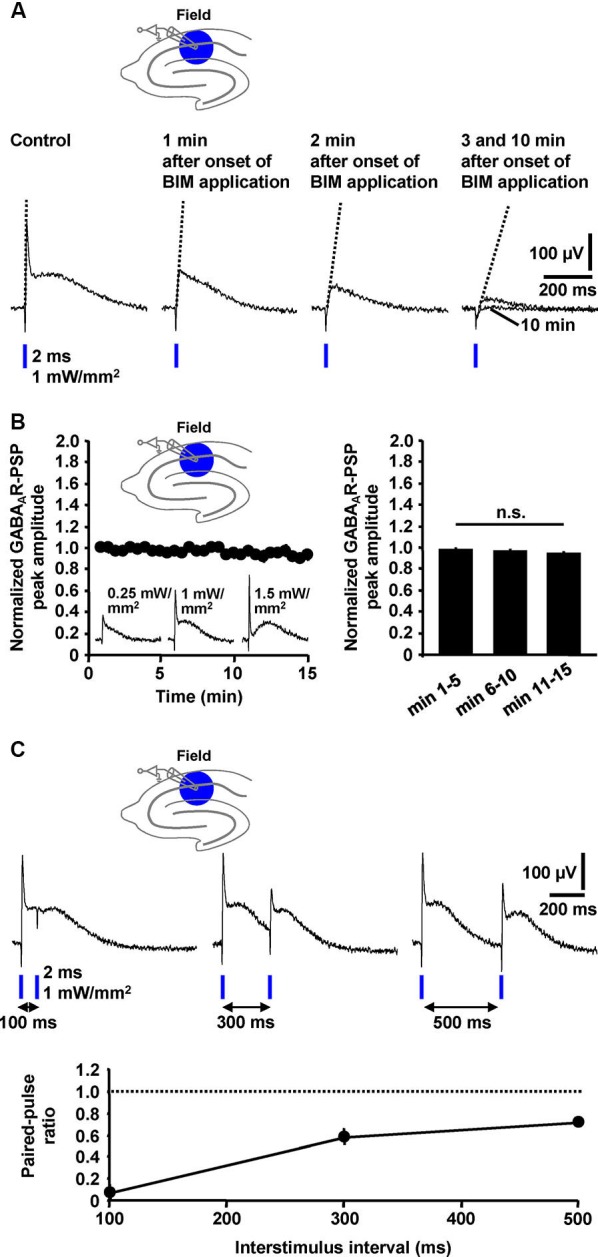
**Quantification, stability, and short-term plasticity of field GABA_A_**R-PSPs. (A) The slope of the rising phase of pharmacologically isolated field GABA_A_R-PSPs (indicated by dotted lines) as well as their peak amplitude and the area under the curve successively decreased during wash-in of BIM to slices, thus making these parameters suited for quantification of the PSPs. **(B**) The peak amplitude of field GABA_A_R-PSPs (evoked by 1 mW/mm^2^ light pulses), which could be increased/decreased by use of higher/lower light intensities (recording traces in left panel), did not significantly change within 15 min of recording (*n* = 5 experiments) [*F*_(2,8)_ = 3.6, *p* > 0.05 (one-way repeated measures ANOVA applied to the data shown in the right panel)]. Data were normalized to the peak amplitude of the first GABA_A_R-PSP evoked. n.s., not statistically significant. **(C)** Field GABA_A_R-PSPs displayed pronounced PPD if two consecutive light pulses were applied at interstimulus intervals of 100, 300, and 500 ms to the CA1 subfield. The paired-pulse ratio was calculated by dividing the value of the peak amplitude of the second GABA_A_R-PSP by the value of the peak amplitude of the first GABA_A_R-PSP. (**B**, **C)** Data are given as mean ± SEM.

Next, we tested whether the peak amplitude of the GABA_A_R-PSP (C2) and/or the slope of its rising phase are suited for quantification. Indeed, during wash-in of BIM (20 µM), this slope of the GABA_A_R-PSP [isolated by means of CGP 55845 (5 µM)] and the amplitude of C2 successively decreased in all experiments performed (*n* = 4) (Figure 4A). Thus, both parameters can be used for quantification. Since it is safe to assume that C4 (Figure 3B1) negatively impacts on these measures, this is, however, only legitimate if the size of C4 does not markedly change during an experiment. Under this condition, the area under the curve appears better applicable for quantification (Figure 4A). All three parameters can also be used for the quantification of pharmacologically isolated GABA_B_R-PSPs (Figure 3B1). We further examined the stability of isolated GABA_A_R-PSPs over many consecutive evocations (30 s interstimulus interval). The peak amplitude of the PSPs, which could be increased/decreased by use of higher/lower light intensities, did not significantly change within the time of recording (15 min) (Figure 4B).

Paired-pulse depression (PPD), a well-characterized form of short-term synaptic plasticity, has been reported to occur at GABAergic synapses onto CA1 pyramidal neurons (Davies et al., 1990). Yet, there is also evidence for paired-pulse facilitation (PPF) at such synapses (Jiang et al., 2000). To explore whether the field GABA_A_R-PSPs display such plasticity and, if so, whether PPD or PPF is the predominant form, we applied blue light pulses at different interstimulus intervals (100, 300, and 500 ms) to the CA1 subfield. In all measurements conducted (*n* = 4), we observed pronounced PPD (Figure 4C).

### Extracellular electrical stimulation in area CA1 failed to induce field GABAR-PSPs

**Figure 5:**
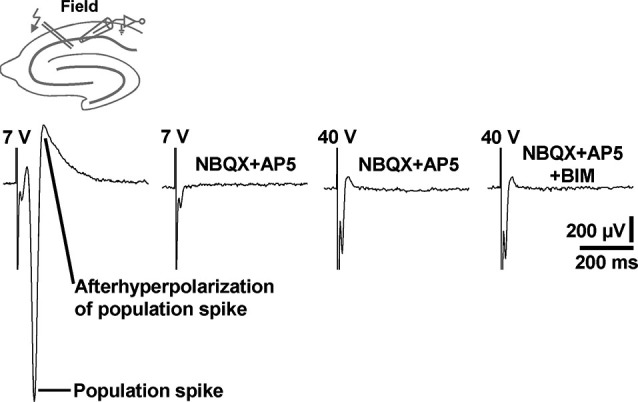
**Electrical stimulation of the Schaffer collateral-commissural pathway failed to evoke CA1 field GABAR-PSPs.** Electrical stimulation pulses delivered to the Schaffer collateral-commissural pathway elicited population spikes in the CA1 cell body layer (first recording trace). The population spikes were fully abolished by coapplication of NBQX and AP5 (second recording trace). Even with markedly higher stimulation intensities, no GABAR-PSPs were observable (third and fourth recording trace). The voltage traces in the presence of NBQX + AP5 and NBQX + AP5 + BIM were acquired 15 min after the onset of NBQX, AP5, and BIM (20 µM) administration. Stimulus artifacts were truncated in part for clarity.

Although being evident from the existing literature, we aimed to demonstrate under our experimental conditions that standard extracellular stimulation paradigms are not appropriate to induce prominent field GABAR-PSPs in area CA1. For this purpose, we performed electrical stimulation of the Schaffer collateral-commissural pathway in slices from C57BL/6N mice and recorded in the transition zone between CA1 stratum pyramidale and CA1 stratum radiatum. Typical for this proceeding (Blank et al., 2002), stimulation pulses of sufficient intensity (7 V) reliably evoked population spikes (*n* = 3 experiments) (Figure 5, first recording trace). Since the population spikes would mask voltage deflections generated by an activation of GABARs, we blocked ionotropic glutamate receptors by addition of NBQX (5 µM) and AP5 (50 µM) to the superfusion medium. This pharmacological treatment always fully abolished the population spikes. Though, even if the stimulation intensity was strongly increased (40 V), we could never detect GABAR-PSPs (Figure 5, second, third, and fourth recording trace). This was also the case if the recording electrode was set to other positions in the transition zone between CA1 stratum pyramidale and CA1 stratum radiatum (data not shown).

### Effect of 5α-THDOC on field GABA_A_R-PSPs

**Figure 6:**
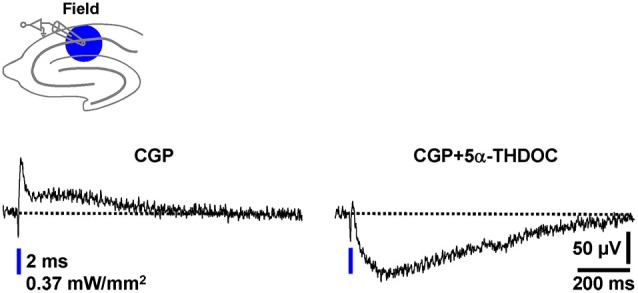
**The neuroactive steroid 5α-THDOC shifts inhibitory field GABA_A_**R-PSPs towards excitatory ones. After recording a stable baseline (30 s interstimulus interval) in the presence of CGP, 5α-THDOC (5 µM) was bath applied to slices. The voltage trace in the presence of the steroid was acquired 15 min after the beginning of its administration.

Natural intracellular ion concentrations, signaling cascades, and pH buffering systems can be crucial for the impact of a particular neuroactive factor on neurotransmission. A striking example for this are the findings that the endogenous steroid 5α-THDOC enhances inhibitory GABA_A_R-mediated responses if neurons are recorded by means of the whole-cell patch-clamp technique (pH buffering by HEPES), but shifts inhibitory GABA_A_R-PSPs towards excitatory ones if alterations of the HCO⁻_3_ gradient across the cytoplasmic membrane are prevented by using sharp microelectrodes for recording (Burg et al., 1998). To potentially further validate the optogenetic assay established here as a useful tool for studying pharmacological manipulations of GABAergic neurotransmission, we finally investigated the effect of 5α-THDOC (5 µM) on pharmacologically isolated field GABA_A_R-PSPs. Indeed, in all experiments conducted (*n* = 4), 5α-THDOC quite rapidly shifted the inhibitory GABA_A_R-PSPs towards excitatory ones (Figure 6).

## Discussion

Optogenetic tools provide a powerful means to dissect the role of distinct neuronal circuits in different types of behavior (Tye et al., 2011; Carter et al., 2013). However, as corroborated here, such tools also can enlarge the spectrum of neurophysiological *in vitro* assays. We employed optogenetic techniques to selectively express ChR2 in forebrain GABAergic cells in mice and demonstrate that, in acute hippocampal slices from the transgenic animals, blue light pulses evoke prominent field inhibitory GABA_A_R- and GABA_B_R-PSPs in area CA1 which are easily and reliably detectable by a single extracellular recording pipette. The field PSPs display typical temporal and pharmacological characteristics, exhibit pronounced PPD, remain stable over many consecutive evocations, and can be quantified by means of standard analysis parameters. We additionally show that the endogenous steroid 5α-THDOC (5 µM) shifts the inhibitory field GABA_A_R-PSPs towards excitatory ones (cf. Burg et al., 1998). Collectively, these findings signify the optogenetic approach established here as a potentially useful tool for future investigations dealing with pharmacological manipulations of GABAergic neurotransmission in mammals. The methodological value of this approach is further underlined by the following facts. First, the hippocampus plays an important role in many brain functions, such as memory formation, spatial navigation, and the regulation of stress responses (Andersen et al., 2007). Second, the hippocampus is associated with severe neurological and psychiatric diseases, such as epilepsy, schizophrenia, and depression (Chang and Lowenstein, 2003; Berton and Nestler, 2006; Lodge and Grace, 2011). Third, the hippocampus is easily accessible for *in vitro* experimentation. And fourth, in contrast to the method described here, intracellular recording approaches are quite expensive, time- and labor-consuming, and, in case of the frequently used whole-cell patch-clamp configuration, affect intracellular ion concentrations, signaling cascades, and pH buffering systems (Burg et al., 1998; Eder et al., 2002; Nugent et al., 2007). However, it is also important to mention that the optogenetic approach established here does not enable the investigation of all aspects of pharmacological effects on GABAergic neurotransmission. These include the modulation of unitary GABAR-PSPs, changes in the conductance of GABA_A_Rs and potassium channels mediating GABA_B_R responses, and modifications of GABAR function induced by intracellularly applied substances. Moreover, with the exception of the paired-pulse paradigm, possible alterations in the probability of presynaptic GABA release cannot be elucidated. This is more directly achievable by the analysis of intracellularly recorded miniature synaptic events. Finally, our optogenetic approach can currently not be used to dissect pharmacological effects on synaptic transmission triggered by specific classes of interneurons. Yet, it appears likely that future studies will be able to do this by expressing ChR2 under the control of e.g., the somatostatin, parvalbumin, or cholecystokinin promoter (Andersen et al., 2007).

Since we also conducted single-cell recordings in the present study, all experiments were performed on an infra-patch setup (Ranft et al., 2007). Yet, for the realization of the optogenetic assay, several of the expensive devices employed here are not necessary. These include the microscope equipped for infrared videomicroscopy (substitutable by a much cheaper binocular), the blue light laser (substitutable by a significantly less expensive LED light source), and the micromanipulator system for the movement of the microscope, the recording chamber, and the intracellular recording electrode (substitutable by a low-cost standard manipulator for field potential pipettes). Moreover, an administration of blockers of glutamatergic neurotransmission (e.g., NBQX and AP5) to the bathing solution (Burg et al., 1998; Eder et al., 2001) is not required. With respect to the guidance of the blue light to the brain slices, a stripped optical fiber can be positioned directly above the neuronal tissue to be illuminated (Bass et al., 2013).

The kinetics of the field GABA_B_R-PSPs (Figure 3B1) fit well to those observed for electrically evoked and intracellularly recorded ones (Pham et al., 1998). This holds also true for the rising phase and initially fast decaying phase of the field GABA_A_R-PSPs (Figure 4A, first recording trace; Burg et al., 1998). However, the field GABA_A_R-PSPs additionally exhibit a slower component (the picrotoxin-sensitive fraction of C3, Figures 3A, B1), the emergence of which can currently not clearly be explained. While it is safe to assume that C4 leads in part to the notch between C2 and C3 (Figures 3A, B1), we can at the moment only speculate that the subsequent GABA_A_R-mediated portion might arise from a spillover of GABA. This could result in a delayed gating of extrasynaptic GABA_A_Rs (Rossi and Hamann, 1998). It is also conceivable that this portion, at least in part, is caused by an activation of ChR2 in GABAergic axon terminals (cf. Tang et al., 2011; Tye et al., 2011). This might additionally trigger an asynchronous (AP-independent) transmitter release into the synaptic cleft. This scenario is supported by the relatively slow inactivation kinetics of ChR2 (Figure 1A2, upper recording trace) and the finding that low-voltage-gated calcium channels, which can actuate GABA release, are present in axon terminals of interneurons forming perisomatic synapses with CA1 pyramidal cells (Tang et al., 2011). Anyhow, the present study provides, to the best of our knowledge, the first demonstration of easily and reliably detectable evoked field GABAR-PSPs in hippocampal slices. It appears likely that the optogenetic approach described here can also be applied to other brain networks abundantly endowed with inhibitory microcircuits (e.g., the central nucleus of the amygdala). It might help to better characterize the impact of e.g., neuroactive steroids and modulators of chloride transporters on GABA_A_R-mediated neurotransmission.

## Conflict of interest statement

The authors declare that the research was conducted in the absence of any commercial or financial relationships that could be construed as a potential conflict of interest.
